# The Role of Autophagy in Glaucomatous Optic Neuropathy

**DOI:** 10.3389/fcell.2020.00121

**Published:** 2020-03-04

**Authors:** Annagrazia Adornetto, Vincenzo Parisi, Luigi Antonio Morrone, Maria Tiziana Corasaniti, Giacinto Bagetta, Paolo Tonin, Rossella Russo

**Affiliations:** ^1^Department of Pharmacy, Health and Nutritional Sciences, Section of Preclinical and Translational Pharmacology, University of Calabria, Rende, Italy; ^2^Visual Neurophysiology and Neurophthalmology Research Unit, IRCCS G.B. Bietti Foundation, Rome, Italy; ^3^Department of Health Sciences, University Magna Graecia of Catanzaro, Catanzaro, Italy; ^4^Regional Center for Serious Brain Injuries, S. Anna Institute, Crotone, Italy

**Keywords:** glaucoma, retinal ganglion cells, autophagy, retina, LC3, p62, CMA, mitophagy

## Abstract

Autophagy is a conserved lysosomal-dependent pathway responsible for the degradation of cytoplasmic macromolecules. Based on the mechanism of cargo delivery to lysosomes, mammalian cells can undergo micro, macro, and chaperone-mediated autophagy. Other than physiological turnover of proteins and organelles, autophagy regulates cellular adaptation to different metabolic states and stressful conditions by allowing cellular survival or, when overactivated, participating to cell death. Due to their structure and function, neurons are highly dependent on autophagy efficiency and dysfunction of the pathway has been associated with neurodegenerative disorders. Glaucomatous optic neuropathies, a leading cause of blindness, are characterized by the progressive loss of a selective population of retinal neurons, i.e., the retinal ganglion cells (RGCs). Here we review the current literature on the role of autophagy in the pathogenic process that leads to the degeneration of RGC in various experimental models of glaucoma exploring the modulation of the pathway as a potential therapeutic intervention.

## Introduction

**FIGURE 1:**
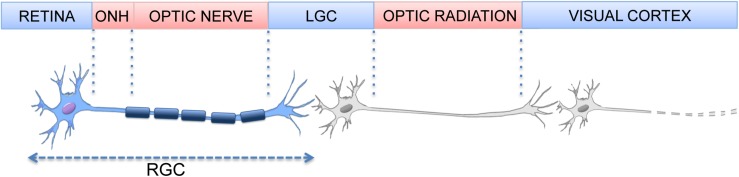
Element of the visual pathway. (Optic nerve Head, ONH; Lateral Geniculate Nucleus, LGN).

Retinal ganglion cells (RGCs) are the last cellular element of the retinal visual pathway and the sole neurons projecting outside the retina; their axons extend through the optic nerve (ON), the chiasm, and optic tract into the midbrain, targeting the superior colliculi and the lateral geniculate nucleus (Erskine and Herrera, 2014; Figure 1). Preserving RGCs function is essential for the transmission of visual information to the central nervous system (CNS) and any damage altering the connection between eye and brain has severe consequences on vision. RGC loss occurs in several ocular pathologies, with glaucoma and diabetic retinopathy being the two major causes (Almasieh et al., 2012).

Glaucoma is a worldwide leading cause of irreversible blindness (Tham et al., 2014). The term comprises of a spectrum of ocular conditions characterized by typical alterations of the ON head and distinct visual field defects. The neurodegenerative process often extends beyond the eye into the lateral geniculate nucleus and visual cortex, suggesting that central areas are involved (Nucci et al., 2015). Age and high intraocular pressure (IOP) are the main risk factors; however, normal tension glaucoma (NTG) occurs in patients with physiological IOP and a portion of patients show progression even if IOP is maintained in the physiological range (Mallick et al., 2016).

Several molecular mechanisms have been involved in RGC apoptosis (excitotoxicity, reduced neurotrophic support, inflammation, etc.) (Russo et al., 2009; Qu et al., 2010). Despite the wealth of preclinical studies showing efficacy for drugs targeting these pathways, almost all failed translation to the clinic, so effective treatment remains a therapeutic challenge.

Several pieces of evidence show that dysfunctional autophagy recurs in neurodegenerative diseases making this process an attractive venue for neuroprotective drug discovery (Menzies et al., 2017). Here we review the current literature linking autophagy with RGC degeneration and the controversial outcomes obtained by manipulating autophagy in experimental glaucoma.

## Autophagy Pathways

Autophagy, a lysosome-mediated degradation system, degrades cytoplasmic macromolecules with the aim to maintain a correct balance in the number of cytoplasmic components. The process is particularly challenging in neurons due to their high metabolic activity, their complex structure, and their inability to dilute toxic protein aggregates by cell division; this sensitivity further increases with aging as a result of reduced autophagy efficiency (Hansen et al., 2018). Based on the mechanism of cargo delivery to lysosomes, three forms of autophagy can be distinguished: macroautophagy, chaperone mediated autophagy (CMA), and microautophagy.

### Macroautophagy

Macroautophagy requires the formation of a double membrane structure that recognizes and engulfs cytoplasmic fragments, proteins, and organelles ending in the formation of a vesicle called autophagosome. Several autophagy-related genes (known as Atg) participate in different steps of macroautophagy; autophagosome formation requires two conjugation events that lead to the formation of the Atg5-12-16 complex and the lipidation of the soluble form of Atg8 (LC3 in mammals), while the initial nucleation step requires a complex formed by Atg6 (beclin-1), AMBRA1, Vps34, Vps15, and Atg14L. Mature autophagosomes fuse with lysosomes, forming the autophagolysosomes; the internal membranes of autophagosomes are degraded together with the cargo, and the degradation products are then released into the cytoplasm and recycled for new processes (Parzych and Klionsky, 2014).

Macroautophagy had initially been thought to be a bulk degradation pathway devoid of selectivity toward its substrates. It has, however, become clear that cargo sorting is present and it is conferred by cytosolic receptor proteins able to tether selective substrates to the nascent autophagosomes, acting as a bridge between the cargo and ATG8-family proteins (Zaffagnini and Martens, 2016). In mammalian cells the autophagy receptor protein binds ubiquitinated substrates; p62/sequestosome 1 (SQSTM1), NBR1, NDP52 and optineurin are four of the so far identified autophagy receptors (Johansen and Lamark, 2011). Furthermore, some macroautophagy processes are selective for the specific degradation of damaged organelles like mitochondria (e.g., mitophagy).

Macroautophagy activity inversely correlates with the activation of the mTOR complex 1 (mTORC1) that, when active, phospho-inhibits the ULK1 complex preventing autophagy (Shang and Wang, 2011). Conversely, the serine/threonine AMP activated kinase (AMPK) promotes autophagy through inhibition of mTORC1 and activation of ULK1 (Egan et al., 2011).

### Mitophagy

Mitophagy mediates the physiological turnover of mitochondria and is responsible for the adaptation of the amount of mitochondria in response to environmental and metabolic changes (Palikaras et al., 2018). As a quality control mechanism, mitophagy is also responsible for the removal of defective mitochondria and it can be induced to inhibit mitochondria-dependent apoptosis (Ney, 2015).

Mitophagy regulatory pathways are distinct in that they are ubiquitination-mediated and mitophagy-receptor-mediated. The PINK-parkin pathway regulates ubiquitin-dependent mitophagy. Under normal conditions, PINK is rapidly cleaved, released into the cytosol, and degraded by proteasome; loss of mitochondrial membrane potential stabilizes PINK at the outer mitochondrial membrane (OMM), recruiting the E3 ligase parkin. In addition to parkin, other ubiquitin E3 ligases (i.e., Gp78, SMURF1, SIAH1, MUL1, and ARIH1) play a role in mitophagy regulation. Once localized on the mitochondrial surface, they generate ubiquitin chains that are recognized by autophagy adaptor proteins (i.e., optineurin, NBR1, and p62) that guide the engulfment of ubiquitinated mitochondria by the autophagosomes (Lazarou et al., 2015).

The receptor-mediated mitophagy relies on proteins localized at the OMM that act as mitophagy receptors and are able to interact with Atg8/LC3. In mammalian cells, OMM proteins NIX, BNIP3, and FUNDC1 – which all have an LC3-interacting region (LIR) – have been defined as mitophagy receptors and are involved in hypoxia-induced mitophagy (Lampert et al., 2019). AMBRA1, a positive regulator of autophagy that stabilizes mTORC1 and the ULK1 complex, is partially localized at the mitochondria and can also act as mitophagy receptor (Cianfanelli et al., 2015). Neurons are highly vulnerable to mitochondrial impairment, and dysfunctional mitophagy has been linked with neurodegenerative conditions like Parkinson’s and Alzheimer’s diseases (Menzies et al., 2017).

### Chaperone-Mediated Autophagy

Chaperone mediated autophagy differs from macro/microautophagy for several reasons: it is highly selective, it does not need the formation of vesicle for lysosomal delivery, single proteins are the only cargo, and they must be recognized by chaperones that mediate their direct translocation through the lysosomal membrane.

The high removal selectivity by CMA involves the formation of complexes between proteins bearing a CMA targeting motif (a pentapeptide sequence similar to KFERQ) and chaperones of the Hsp70 family; these complexes are recognized by the lysosome-associated membrane protein type-2A (LAMP2A) at the lysosomal membrane where the substrate proteins unfold and translocate in the lumen and are degraded by lysosomal hydrolase. Proteins that do not carry the KFERQ motif can also be degraded by CMA after post-translational modifications (Dice, 2007). LAMP2A acts as a limiting element for the activity of CMA, and changes in LAMP2A levels correlate with CMA activity (Cuervo and Dice, 2000).

Basal CMA can be detected in almost all mammalian cells, although each tissue shows a different level, suggesting that dependency on this pathway varies among cell types (Koga et al., 2011). As part of the cellular response to stressful conditions, CMA can be further induced by a variety of stimuli, like hypoxia (Hubbi et al., 2013) and oxidative stress (Kiffin et al., 2004).

## Autophagy and RGC Degeneration

Elevated IOP can cause direct mechanical stress to RGC axons together with ischemic effects due to the compression of the blood vessels supplying ON head. Furthermore, it might cause damage to the dorsal attachment of astrocytes at the ON head, causing loss of metabolic support and mechanical strength to RGC axons (Dai et al., 2012).

Increased LC3 immunoreactivity at the ganglion cell layer (GCL) and accumulation of LC3II has been shown between 6 and 24 h after a transient IOP increase in rats (Piras et al., 2011; Produit-Zengaffinen et al., 2014; Wei et al., 2015). In this same model, evidence of autophagy impairment due to calpain-mediated cleavage of beclin-1 was reported in the insulted retina (Russo et al., 2011).

The mice subjected to transient retinal ischemia in our group have shown a biphasic, reperfusion-time dependent modulation of autophagy. Autophagic flux experiments demonstrated that, in the injured retina, autophagy induction reached a peak 6 h after the insult, while autophagic flux reduced after 24 h; declined autophagic turnover was suggested by a decrease of ATG proteins (ATG4, ATG12-5, BECN1) and accumulation of p62-positive bodies in RGC cytoplasm (Russo et al., 2018). In this study, increased RGC loss was observed in mice with genetic impairment of basal autophagy due to the heterozygous ablation of AMBRA1 (Russo et al., 2018).

Impairment of the autophagic flux with an accumulation of p62 and autophagic vacuoles has been reported in RGC axons following chronic IOP elevation by laser photocoagulation (Kitaoka et al., 2013). Similarly, an increase of autophagic vesicles was shown in the retina of chronic hypertensive rhesus monkeys; however, upregulation of lysosomal activity and an increase of LC3II and beclin-1 expression suggested activation, rather than impairment, of the autophagic flux (Deng et al., 2013).

Autophagy was significantly activated in a model of chronic hypertensive glaucoma induced by episcleral veins cauterization (EVC), as demonstrated by an accumulation of autophagosomes in the soma and dendrites of RGCs and upregulation of beclin-1 and LC3II/LC3I ratio. Inhibition of autophagy by 3-methyladenine (3-MA) decreased the number of apoptotic neurons in GCL, suggesting that autophagy induced cell death (Park et al., 2012, 2018). By contrast, after IOP elevation by laser photocoagulation, 3-MA exaggerated axonal degeneration, while the mTOR inhibitor rapamycin reduced axonal accumulation of p62 and exerted axonal protection against ON degeneration (Kitaoka et al., 2013).

Our group has recently shown that subchronic systemic treatment with rapamycin sustained autophagy in retinas subjected to a transient increase of IOP and reduced RGC loss (Russo et al., 2018). Accordingly, pharmacological inhibition of mTOR afforded neuroprotection to RGCs following chronic ocular hypertension and suppressed apoptosis of primary RGCs induced by glutamate (Su et al., 2014). Conversely, an acute systemic administration of rapamycin exacerbated RGC death induced by ON ischemia (Chen et al., 2016) and deletion of the negative regulator of mTOR, i.e., PTEN, in RGCs promoted ON regeneration and increased RGC survival after ON crush (Park et al., 2008).

Following ON transection, a model mimicking the impairment of retrograde axonal transport of trophic factors occurring in glaucoma, upregulation of beclin-1 and LC3II expression was reported in whole retinas and isolated RGCs (Kim et al., 2008). A subsequent study showed that autophagy is activated in axotomized retinas and suggested a cytoprotective role of this pathway; indeed, genetic downregulation of autophagy, by RGC-specific deletion of Atg5 or Atg4B knocking down, was associated with reduced RGC survival, while rapamycin treatment increased the number of surviving RGCs (Rodriguez-Muela et al., 2012).

An opposite conclusion was drawn by Koch and colleagues that showed a post-lesional Ca^2+^-dependent activation of autophagy with an accumulation of autophagosomes in RGC axons following ON crush; in this study, pharmacological inhibition of autophagy by 3-MA delayed axonal degradation (Koch et al., 2010).

Recently, a decrease of LC3II/LC3I ratio and increase of p62 after ON crush was shown to imply an impairment of the autophagy process in this model (Oku et al., 2019). p62 siRNA facilitates autophagy by preventing p62 aggregates accumulation (Kojima et al., 2014); in a rat model of ON crush p62 siRNA offered better preservation of visual function and greater prevention of RGC apoptosis as compared to rapamycin treatment. This was mediated by a specific deactivation of mTORC1 without inhibition of mTORC2-mediated positive effects (i.e., Akt phosphorylation, stabilization of the blood ocular barrier, M2 macrophage polarization) (Wen et al., 2019).

In DBA/2J mice, a spontaneous ocular hypertensive model of glaucoma, an increased number of autophagosomes, higher LC3II/LC3I, and decreased LAMP1 were observed in myelinated ON axons. This observation was associated with increased mitochondrial damage and no changes in PINK or parkin levels, suggesting that the degenerating organelles may not be efficiently removed by mitophagy (Coughlin et al., 2015).

More recently, Hirt and colleagues monitored autophagy in the iridocorneal angle region and retina of DBA/2J and transgenic DBA/2J:GFP-LC3 mice; in both strains decreased LAMP1 and upregulation of LC3II and p62 were reported in the angle region, while decreased levels of all three proteins were shown in the RGC bodies, suggesting an overall slowed autophagic flux (Hirt et al., 2018).

Mutation of the optineurin genes has been associated with an autosomal dominant form of hereditary NTG (Rezaie et al., 2002). Optineurin is recruited to damaged mitochondria following parkin and, subsequently, recruits LC3 resulting in autophagosomes formation around mitochondria. Alteration of these latter steps, as it has been shown for an ALS-associated optineurin mutant, leads to inefficient PINK-Parkin-associated mitophagy and pathological accumulation of damaged mitochondria (Wong and Holzbaur, 2014). The E50K mutation is the most common optineurin mutation associated to NTG; increased LC3 immunoreactivity, detection of autophagosome, and autolysosome were reported in RGCs of E50K transgenic mice (Shen et al., 2011). Furthermore, E50K altered mitochondrial dynamics and induced mitochondrial fission and mitophagy in the axon of glial lamina of aged E50K transgenic mice and cultured primary RGCs (Shim et al., 2016).

Upregulation of parkin and optineurin was reported in the GCL of hypertensive rats; in this model, the number of unhealthy mitochondria and mitophagosomes were increased and it was associated with a higher LC3II to LC3I ratio and decreased LAMP1 two weeks following IOP elevation, suggesting a reduction of autophagosome turnover and local mitochondrial recycling (Dai et al., 2018). Overexpression of parkin reduced RGC loss, promoted optineurin expression, and partially restored mitophagy dysfunction in the ON (Dai et al., 2018). The mitochondrial uncoupling protein 2 (UCP2) reduces mitochondrial oxidative stress and exerts neuroprotective effects by uncoupling oxygen consumption from ATP synthesis (Richard et al., 2001). Deletion of UCP2 reduced RGC death in mice with chronic elevated IOP by increasing the level of mitophagy and facilitating mitochondrial function (Hass and Barnstable, 2019).

## Trabecular Meshwork Autophagic Deregulation in Glaucoma

Trabecular meshwork (TM), a tissue involved in maintaining IOP into physiological range, is constantly exposed to stresses such as oxidative, mechanical, and shear stress that, in the long term, may alter tissue function leading to higher resistance in aqueous humor outflow often associated with glaucoma. Porter and colleagues demonstrated that autophagy is induced in chronically stressed TM cells, however, the concomitant reduced acidification of the lysosomal compartment, improper cathepsin maturation, and decreased cathepsin B activity resulted in reduced autophagic flux (Porter et al., 2013). This same group compared the autophagic function of TM cells isolated from glaucomatous and age-matched donor eyes showing a mTOR-dependent dysregulation of autophagic pathway (i.e., lower basal levels of LC3II and p62) and autophagy response in glaucomatous TM cells (Porter et al., 2015). A recent study on human TM cells exposed to chronic oxidative stress reported protective effects of rapamycin that were associated with removal of damaged mitochondria, suggesting a protective role for autophagy in TM cells (He et al., 2019).

Autophagy is also activated in TM cells exposed to biaxial mechanical stretch and high pressure in a mTOR and BAG3 independent manner; interestingly, cyclic, but not static, mechanical stimulation of TM cells seems to activate a chaperone-assisted selective autophagy (CASA) (Hirt and Liton, 2017), a selective tension-induced autophagy previously described in muscle (Ulbricht et al., 2013).

## The Interplay Between Macroautophagy and CMA

Macroautophagy and CMA are both essential components of the stress response in mammalian cells and several studies suggest a reciprocal interplay so that changes in the activity of one will affect the contribution of the other to protein degradation. For instance, blockade of macroautophagy, both pharmacologically or knocking down Atg5 gene, results in CMA up-regulation under normal and stressed nutritional conditions (Kaushik et al., 2008). Furthermore, in several experimental systems, a sequential, rather than a concomitant, activation of these two forms of autophagy can be observed. For example, in liver and cultured fibroblasts, macroautophagy is activated during the first hours of starvation, while CMA takes over at later time points (Cuervo et al., 1995; Massey et al., 2006). An inverse order of activation has been reported in case of presence of misfolded proteins: CMA is activated first to degrade the substrate and, subsequently, macroautophagy comes into place to remove the multimeric complexes formed by the misfolded proteins (Ravikumar et al., 2002; Iwata et al., 2005).

The requirements for active CMA or the possibility to replace it with macroautophagy depends on the insult, the type of protein damage (aggregation *versus* unfolding), and the type of substrates (protein *versus* organelles). This implies that CMA and macroautophagy cannot completely compensate for each other’s function.

However, this interplay between macroautophagy and CMA seems to be cell-type dependent. Indeed, in aged retinas and Atg5 deficient mice reduced activity of macroautophagy was accompanied by upregulation of the CMA markers LAMP2A and Hsp70; however, *in vitro* downregulation of CMA did not elicit macroautophagy activation in photoreceptor cells (Rodriguez-Muela et al., 2013).

## Discussion

The controversial results reported on the role of autophagy in insulted RGCs, with autophagy either protecting or promoting cell death (see Table 1), still leaves several questions on the clinical feasibility of targeting autophagy to achieve retinal neuroprotection. Interpretation of the results under an integrated hypothesis is further complicated by the use of different animal models, each mimicking a single aspect of the disease (i.e., hypoxic events, alteration of neurotrophin transportation, acute or chronic hypertension). Some of the answers may reside in the spatio-temporal regulation of the process and the interplay between the autophagy subtypes. Indeed, with autophagy being a dynamic process, the results should be interpreted considering the time selected after the initial insult and the neuronal compartment analyzed (axon *versus* soma). Another drawback of many publications is the lack of autophagic flux assay that limits the correct interpretation of the data (Klionsky et al., 2016). It must also be considered that several studies used *in vitro* and *in vivo* models expressing GFP-LC3, this can form aggregates that are often indistinguishable from autophagosomes by fluorescence microscopy and may also alter the physiological autophagosome turnover (Kuma et al., 2007; Hirt et al., 2018). Moreover, the available drugs modulating autophagy affect multiple pathways, making the isolation and interpretation of autophagy in the different experimental settings difficult.

**TABLE 1:** Autophagy modulation in experimental models of glaucoma.

Experimental model		Analyzed structure	Autophagy markers	Effects of autophagy modulators	References
Retinal ischemia/reperfusion	Male wistar rats	Retina	↑ LC3II, ↑ autophagosomes	3-MA prevents neuronal loss in GCL and reduces apoptotic markers	Piras et al., 2011
	Male Sprague-Dawley Rats	Retina	↑ LC3II (3 h)	Rapamycin increases the number of apoptotic cells in GCL	Produit-Zengaffinen et al., 2014
	Male Sprague-Dawley Rats	Retina	↑ LC3II, ↑ autophagosomes		Wei et al., 2015
	Male wistar Rats	Retina	↓ LC3II, ↓ beclin-1		Russo et al., 2011
	Male C57BL/6J, Ambra1^+/gt31^, GFP-LC3 Mice	Retina	↑ LC3II (6h), ↓ LC3II (24 h) ↑ p62	Rapamycin and fasting reduce RGC loss Ablation of AMBRA1 increases RGC loss	Russo et al., 2018
Laser photocoagulation	Male wistar rats	Optic nerve	↑ LC3II, ↑ p62, ↑ autophagosomes	3-MA increases axonal degeneration Rapamycin reduces axonal degeneration	Kitaoka et al., 2013
	Male and Female Rhesus Monkey	Retina	↑ LC3II, ↑ LC3II/LC3I, ↑ beclin-1 ↑ autophagosomes		Deng et al., 2013
Episcleral veins cauterization	Male Sprague-Dawley Rats	Retina	↑ LC3II, ↑ LC3II/LC3I, ↑ beclin-1	3-MA prevents neuronal loss in GCL	Park et al., 2012
	Male Sprague-Dawley Rats	Retina	↑ LC3II/LC3I, ↑ beclin-1	3-MA prevents RGC apoptosis	Park et al., 2018
	Sprague-Dawley Rats	Retina, Primary RGCs		Rapamycin prevents RGC loss	Su et al., 2014
Optic nerve transection	Male wistar rats	Retina, Primary RGCs	↑ mRNA Atg5, Atg7, Atg12 ↑ beclin-1, ↑ LC3II (3 h)		Kim et al., 2008
	GFP-LC3 Mice, Atg4B^–/–^ Mice, Atg5 ^flox/flox^ Mice	Retina	↑ LC3II, ↑ autophagosomes	Rapamycin reduces RGC death	Rodriguez-Muela et al., 2012
Optic nerve crush	Female wistar rats	Optic nerve	↑ autophagosomes	3-MA delays axonal degradation	Koch et al., 2010
	Male wistar rats	Retina	↓ LC3II/LC3I, ↑ p62		Oku et al., 2019
	Male wistar rats	Retina	↑ mRNA p62, ↑ LC3II, ↑ LAMP1	p62 siRNA and rapamycin prevents RGC apoptosis	Wen et al., 2019
DBA/2J Mice		Optic nerve	↑ LC3II/LC3I, ↓ LAMP1, ↑ autophagosomes		Coughlin et al., 2015
		Retina	↑ LC3II, ↑ p62, ↓ LAMP1 in angle region		Hirt et al., 2018
			↓ LC3II, ↓ p62, ↓ LAMP1 in RGC bodies		

Although it is clear that modulation of autophagy represents a consistent response of RGCs to detrimental insults, the availability of selective autophagy modulators and a detailed understanding of the contribution of the different autophagy pathways are required for future translation of experimental data into glaucoma therapy.

## Author Contributions

AA and RR wrote the manuscript. RR, LM, and PT edited and reviewed the manuscript. VP, GB, and MC acquired the financial support.

## Conflict of Interest

The authors declare that the research was conducted in the absence of any commercial or financial relationships that could be construed as a potential conflict of interest.
